# *Thainema* gen. nov. (Leptolyngbyaceae, Synechococcales): A new genus of simple trichal cyanobacteria isolated from a solar saltern environment in Thailand

**DOI:** 10.1371/journal.pone.0261682

**Published:** 2022-01-07

**Authors:** Somayeh Rasouli-Dogaheh, Jiří Komárek, Thomrat Chatchawan, Tomáš Hauer

**Affiliations:** 1 Department of Botany, Faculty of Science, University of South Bohemia, České Budějovice, Czech Republic; 2 Department of Biology, Faculty of Science, Chiang Mai University, Chiang Mai, Thailand; National Cheng Kung University, TAIWAN

## Abstract

Simple trichal types constitute a group of cyanobacteria with an abundance of novel, often cryptic taxa. Here, we investigated material collected from wet surface-soil in a saline environment in Petchaburi Province, central Thailand. A morphological comparison of the isolated strain with similar known species, as well as its phylogenetic and species delimitation analyses based on the combined datasets of other related organisms, especially simple trichal cyanobacteria, revealed that the material of this study represented an independent taxon. Using a multifaceted method, we propose that this material represents a new genus, *Thainema* gen. nov., belonging to the family Leptolyngbyaceae, with the type species *Thainema salinarum* sp. nov. This novel taxon shares similar ecological habitats with strains previously placed in the same lineage.

## Introduction

Cyanobacteria are ubiquitous microorganisms abundantly found in a wide range of terrestrial and aquatic habitats [1]. These microorganisms played a crucial role in the evolution of life on Earth by producing and releasing oxygen into the atmosphere, starting approximately 2.5 billion years ago [2]. Cyanobacteria are one of the most abundant microorganisms and are adequately adapted to extreme environments, such as solar saltern environments, where salt concentrations are higher than that in seawater [3, 4]. Cyanobacteria show high tolerance and adaptability to harsh environmental conditions because of their ability to withstand high osmotic pressure, probably as a result of their long evolutionary history [5, 6].

Notwithstanding the ecological and evolutionary importance of cyanobacteria, their systematics has significant gaps. These problems are intensified by cultivation difficulties, reduced sampling from regions outside the temperate zone, and challenging species concepts [7–10]. On the other hand, cyanobacteria are a monophyletic, but morphologically diverse, group [11, 12]. Moreover, the traditional classification of cyanobacteria, based on morphological features, takes no notice of cryptic taxa (e.g., [13, 14]).

Molecular phylogenetic analysis has become a powerful approach in modern taxonomy and has been used to elucidate the evolutionary patterns of cyanobacteria [15–17]. Taxonomic delineation of organisms based on the 16S rRNA gene improves their classification and is recognized as the most commonly used molecular marker-based method for the identification of prokaryotes [9, 18]. In addition, predictions of the secondary structure of the 16S-23S internal transcribed spacer (ITS) region are regarded as a fundamental tool for the speciation of cyanobacteria [19–23]. However, the identification of many taxa within some cyanobacterial orders remains difficult, and further research is needed to better elucidate the relationships among their members. Although some approaches for species identification are rarely used for cyanobacteria, they are increasingly being used for algae and cyanolichens (e.g., [24, 25]). Species delimitation analyses using tree-based and non-tree-based methods have enabled the identification of novel species, and are being utilized as a substantive concept in current systematics [26, 27].

Synechococcales is a large, polyphyletic order comprising more than 90 genera of both unicellular and filamentous cyanobacteria; however, most of the families and genera within the order have not been revised by modern phylogenetic studies [9]. For example, Leptolyngbyaceae, one of the largest families in the order Synechococcales, has been investigated more extensively than any other family in this order. Most of the genera in the Leptolyngbyaceae family are monophyletic, with some exceptions that await precise polyphasic analyses (see reviews [9, 14, 19, 28–34]).

A large number of genetic and morphological studies have been conducted to date, with a focus on the taxonomy of Leptolyngbyaceae (e.g., [29, 30, 35, 36]). Li & Li [35] transferred five strains of *Planktolyngbya circumcreta* to the new genus *Limnolyngbya* within the Leptolyngbyaceae family. However, within this family, the general systematic patterns of the genera have yet to be clarified. *Halomicronema*, one of the most well-known filamentous taxa of microbial mats in extreme hypersaline habitats, grows in high salt concentrations ranging from 7% to 15% [37, 38]. *Halomicronema* sp. was morphologically identified by Chatchawan et al. [39] in solar saltern environments. However, some of morphological characters of this strain were not consistent with the type species *Halomicronema excentricum* Abed et al. [37]; therefore, they suggested molecular analysis for a detailed validation [39].

In this study, we focused on cyanobacterial matter isolated from a manmade solar saltern environment. This material, as cited above, was first reported and morphologically characterized by Chatchawan et al. [39] under the name of *Halomicronema* sp. We sought to (1) perform additional morphological analysis of this material, including ultrastructure analysis; (2) conduct its molecular characterization as well as an analysis of the phylogenetic relationships of the strains with the other members of the Synechococcales order; and (3) determine the evolutionary lineages and species delimitation of *Halomicronema* sp. using both tree- and genetic distance-based approaches. Based on the results of this study, we propose that this material is a new genus within the Leptolyngbyaceae family.

## Material and methods

### Collection site and strain isolation

The source material was collected by Chatchawan et al. [39] from wet soil in shallow evaporation basins in the Ban Laem district, Petchaburi Province, Thailand (13.30 N, 100.07 E), in November–December 2009. After sampling, a portion of soil sample was transferred onto BG-11 agar medium containing different salt concentrations, with the soil spread throughout the medium and the cultures maintained under the following conditions: 25°C, 12 h light/12 h dark cycle, and 28 μmol·m^2^·s^–1^ light intensity. The cyanobacterial species were isolated, transferred onto new agar medium, and maintained until monospecific strains were acquired [39]. The strains were maintained as a private collection at the Institute of Botany AS CR, Třeboň, Czech Republic. Subsequently, the strain was deposited into the CCALA culture collection (Třeboň, Czech Republic) under the accession number CCALA 10287. Additionally, the UTEX B SP44 *Pseudanabaena galeata* strain was obtained from the UTEX culture collection and compared with the material collected in this study.

### Microscopic investigation

The morphological characteristics of the isolated cyanobacterial strain were analyzed using the Olympus BX 53 light microscope (LM) and identified according to Komárek & Anagnostidis [40].

To conduct the transmission electron microscopy analysis of the isolated strain, the cells were preserved in a mixture of 2.5% glutaraldehyde and 0.1 M cacodylate buffer, and then washed with the same buffer. Subsequently, the cells were postfixed in 2% osmium tetroxide, dehydrated using an acetone dilution series, and then embedded in Spurr’s resin [41]. Thin sections were cut using a diamond knife, placed on Formvar coated grids, contrast-treated with uranyl acetate and lead citrate, and then coated with carbon. The sections were observed with a JEOL 1010 transmission electron microscope (TEM).

### DNA isolation, PCR amplification, and sequencing

The strain biomass was dried for 48 h over silica gel, and powdered in a Mixer Mill MM200 (Retsch, Haan, Germany). Genomic DNA (gDNA) was extracted from the dried biomass using an UltraClean Microbial DNA Isolation Kit (MO BIO Laboratories, Carlsbad, CA, USA). Sequences of the 16S rRNA gene, the 16S-23S ITS region [42, 43], *rpoC1* [44], and *rbcLX* [45] were amplified by polymerase chain reaction (PCR) (S1 and S2 Tables) in 20 μl reactions, each containing 1 μl gDNA, 0.6 μl of each primer (10 pmol·μl^-1^), 10 μl of 2X Plain Combi PP Master Mix (1 U Hot Start Taq polymerase in the manufacturer’s reaction buffer, 2.5 mM MgCl_2_, and 0.2 mM of each dNTP [Top-Bio, Prague, Czech Republic]), and 7.8 μl sterile water. The PCR products were separated from the primer dimers and residual gDNA by electrophoresis at 60 V for 45 min using 1.5% low melting point agarose gel. The PCR products were cloned into the pGEM-T Easy (Promega, Madison, WI, USA) vector and transformed into *Escherichia coli*. Plasmids were purified from *E*. *coli* using the NucleoSpin Plasmid Kit (Macherey-Nagel, Düren, Germany), and sequenced using universal primers, T7 (5’-TAA TAC GAC TCA CTA TAG GG-3’) and SP6 (5’-TAT TTA GGT GAC ACT ATA G-3’), to independently obtain sequences of both strands [46].

Four plasmids were sequenced for strain CCALA 10287, whereas three plasmids were sequenced for strain UTEX SP44. The sequences were submitted to the National Center for Biotechnology Information (NCBI) GenBank database (www.ncbi.nlm.nih.gov) (S3 Table).

### Sequence and phylogenetic analyses

Sequences of the cloned PCR products were merged using BioEdit version 7.2.5 [47] and SeqScape version 4.0 (Applied Biosystems), and then compared with sequences available in the NCBI database using BLASTn (http://www.ncbi.nlm.nih.gov/BLAST). The new sequences were aligned to 129 sequences from the NCBI database (S3 Table) using MAFFT version 7 [48], with default settings. *Gloeobacter violaceus* was designated as an outgroup. An uncorrected pairwise genetic distance (P-distance) for 16S rRNA was estimated with 1,000 bootstrap replicates in MEGA 6 [49] using (100 x (1-genetic distance)). Genetic distance within the members of the newly described genus and between all groups was calculated based on the uncorrected pairwise genetic distance with 1,000 bootstrap replicates using MEGA version 6. The multilocus datasets of 16S rRNA (1,160 bp), *rpoC1* (871 bp), and *rbcLX* (777 bp) genes, and the ITS region (421 bp) were concatenated. PartitionFinder 2.1 [50] was used to recognize the best model of sequence evolution for each partition using the “greedy” algorithm and Bayesian Information Criterion (BIC).

To evaluate the relative support of the branches, Maximum Likelihood (ML) analysis was accomplished using the IQ-TREE web server [51]. A total of 1,000 bootstrap pseudoreplications were run to evaluate the relative support of the branches [52]. Bayesian Inference (BI) analyses involved two runs of eight Markov chains, executed for 60 million generations using default parameters and sampled after every 1,000 generations. The final average standard deviation of split frequencies was <0.01, and the first 25% of the sampled data was discarded as burn-in. Convergences were checked using Tracer version 1.7 [53], and phylogenetic trees were redrawn in FigTree version 1.4.4 [54].

### Molecular diagnosis of families

Five conserved regions (helices 18, 20, 23, 27, and 34) of the 16S rRNA gene sequence were selected for family-level identification of the collected material, based on comparisons with the same regions of other related taxa (type species or reference sequences), and two regions (helices 23 and 27) were chosen for secondary structure comparisons [14, 55].

### Species delimitation

Four species-boundary analyses were performed to evaluate the theoretical species boundaries in the 16S rRNA dataset.

#### Genetic distance-based and secondary structure analyses

The ‘barcoding gap’ concept was used to identify the strain at the species level using the 16S rRNA dataset. Automatic Barcode Gap Discovery (ABGD) analysis (http://wwwabi.snv.jussieu.fr/public/abgd/abgdweb.html) was run to detect barcode gaps in the pairwise genetic distance of the dataset. The maximum value of the prior intraspecific divergence was set between 0.001 and 0.01.

The secondary structure of the 16S-23S rRNA ITS region (including D1-D1´ and Box-B helices) was folded using the RNA Secondary Structure Prediction Web Server (https://rna.urmc.rochester.edu/RNAstructureWeb), and then redrawn in CorelDRAW Graphics Suite 2018.

#### Tree-based analyses

If the putative species limits are unknown, the Generalized Mixed Yule Coalescent (GMYC) approach can be used to estimate the species boundaries. The likelihood framework in GMYC allows for statistical inference and hypothesis testing across the entire clade, which is beneficial. The GMYC analyses were carried out using the *ape*, *gee*, *MASS*, *paran*, and *splits* packages of the R statistical program. The GMYC approach, which uses an ultrametric gene tree reconstructed in Beast 1.8.2 using a clock model [56], was applied using an uncorrelated lognormal relaxed molecular clock as a model. The BIC, which is implemented in PartitionFinder 2.1.1, was used to find the best substitution model [50]. The analysis was executed under the constant population size coalescent, as the tree prior, and the Ucld.mean parameter was set prior to exponential distribution, with a mean of 10 and an initial value of two independent chains for 600 million generations, with sampling every 10,000^th^ generation, and with the removal of the first 25% of burn-in trees. Tracer version 1.7 [53] was used to check the outputs for convergence, and TreeAnnotator 1.8.2 was used to generate the consensus tree [56]. The ML of the GMYC model was tested using a likelihood ratio test against a null model that treated the entire tree as a single coalescent (i.e., against a one-species model).

The Bayesian version of Poisson Tree Processes (bPTP) analysis, based on the MrBayes consensus tree, was also run for delimiting species on a rooted phylogenetic tree. The analysis was carried out on the bPTP web server (http://species.h-its.org/ptp/) for 600,000 generations using 0.3 burn-in and 100 thinning, and both Bayesian and ML algorithms for bPTP were considered.

## Results

### Phylogenetic analysis

**Fig 1:**
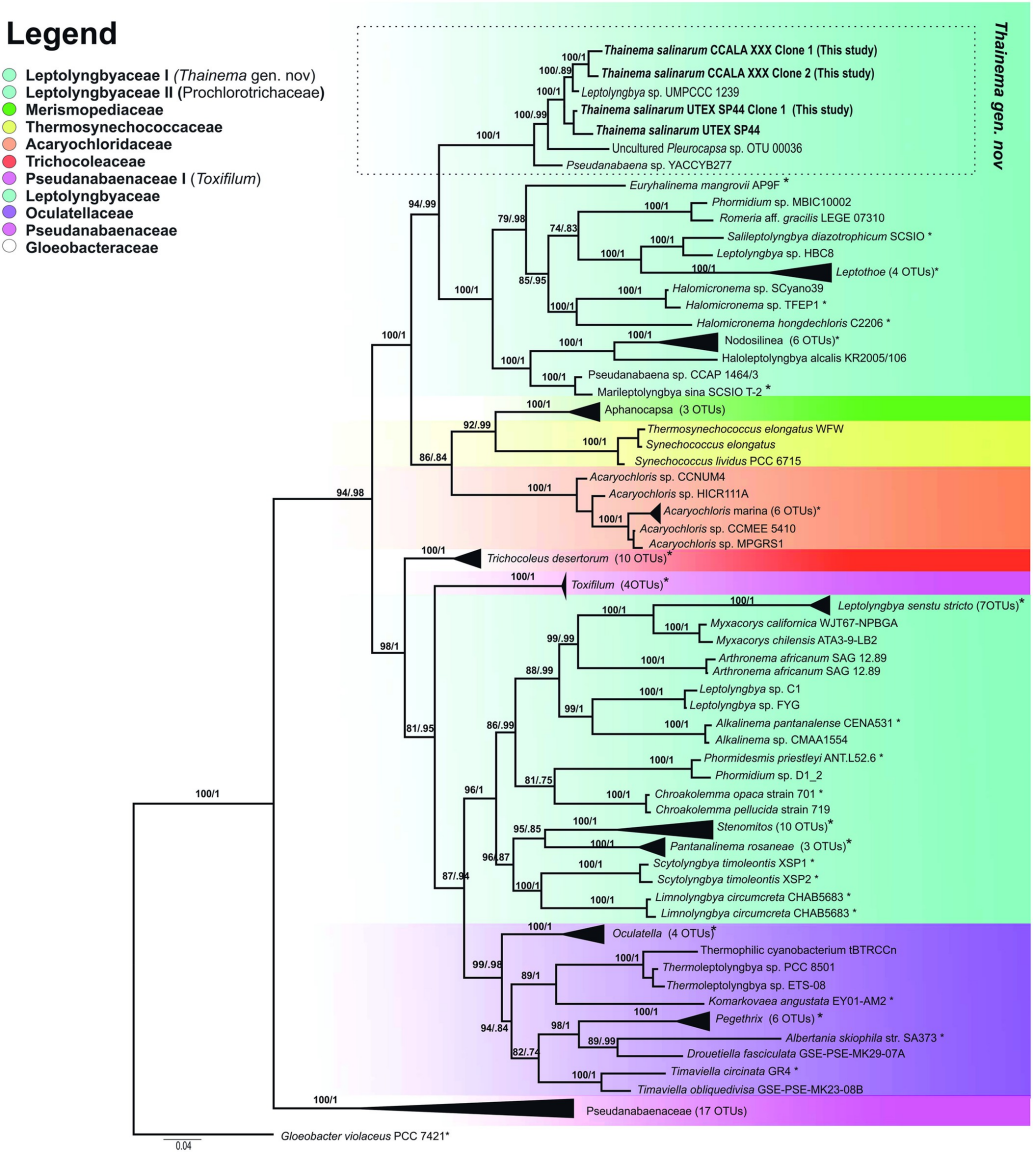
Phylogenetic analysis based on a multilocus dataset, showing the position of the newly described genus (*Thainema* gen. nov.). The tree is based on Bayesian topology, and the support values are given for both the Bayesian posterior probabilities plus the bootstrap values for the maximum likelihood tree. The scale bar represents the number of nucleotide substitutions per site. Reference sequences of the taxa are marked with an asterisk (*). The legend indicates the families and previously proposed family in the tree.

A phylogenetic tree was constructed based on a multilocus (16S rRNA, 16S-23S ITS, *rpoC1*, and *rbcLX*) dataset of 3,229 bp. The results revealed at least 10 different lineages in the order Synechococcales (Fig 1). The phylogenetic trees contained the new clade identified in this study (*Thainema* gen. nov.), *Leptolyngbya* sensu stricto, several newly established and revised taxa (*Euryhalinema*, *Salileptolyngbya*, *Leptothoe*, *Romeria*, *Nodosilinea*, *Haloleptolyngbya*, *Halomicronema*, *Acaryochloris*, *Aphanocapsa*, *Thermosynechococcus*, *Toxifilum*, *Trichocoleus*, *Myxacorys*, *Arthronema*, *Alkalinema*, *Phormidesmis*, *Chroakolemma*, *Stenomitos*, *Pantanalinema*, *Scytolyngbya*, *Limnolyngbya*, *Oculatella*, *Thermoleptolyngbya*, *Komarkovaea*, *Drouetiella*, *Albertania*, and *Timaviella*), and some members of Pseudanabaenaceae and other related taxa belonging to Synechococcales. The tree contained some strains that had previously been incorrectly placed taxonomically, mostly in *Leptolyngbya* and *Pseudanabaena* at the base of the Synechococcales. The new clade (*Thainema* gen. nov.) was separate from its sister clade, and contained the taxa *Euryhalinema*, *Salileptolyngbya*, *Marileptolyngbya*, *Romeria*, *Leptothoe*, *Nodosilinea*, and *Halomicronema*, with the highest support values (bootstrap support [BS] = 94; posterior probability [PP] = 0.99). Collectively, *Thainema* gen. nov. and its sister clade were separated from the Acaryochloridaceae, Merismopediaceae, and Thermosynechococcaceae clades with strong support values (BS = 100; PP = 1). The clades mentioned above were separated from the whole dataset with the highest support values (BS = 97; PP = 0.98). The phylogenetic tree showed that Leptolyngbyaceae and Pseudanabaenaceae families exhibit a polyphyletic structure within the order.

### Analysis of 16S rRNA sequence similarity for genus- and family-level separation

Tables 1 and 2 show the sequence similarity of the 16S rRNA gene within the *Thainema* gen. nov. members and between other phylogenetically related groups in the tree, respectively. The highest and lowest values of sequence similarity between groups were estimated to be 93.5% for Trichocoleaceae and 90.2% for Leptolyngbyaceae respectively. The average similarity in the 16S rRNA gene sequences between families was 92.1%.

**Table 1:** 16S rRNA dissimilarity among *Thainema* strains (displayed values are in %).

	***Thainema gen. nov***.	***1***	***2***	***3***	***4***	***5***	***6***	***7***
***1***	*Leptolyngbya* sp._UMPCCC 1239 KM218876							
***2***	Uncultured *Pleurocapsa* sp. 00036 KM462585	2.02						
***3***	*Pseudanabaena* sp. YACCYB27_MH683727	1.5	2.29					
***4***	***Thainema salinarum* CCALA 10287_clone1_This study**	0.64	1.93	1.83				
***5***	***Thainema salinarum* CCALA 10287_clone2_This study**	0.55	2.02	1.74	0.45			
***6***	***Thainema salinarum* UTEX_SP44 HQ658458**	1.52	3.26	2.66	1.67	1.57		
***7***	***Thainema salinarum* UTEX_SP44_clone1_This study**	0.44	2.06	1.42	0.71	0.62	1.14	

**Table 2:** 16S rRNA genetic similarity among the *Thainema* gen. nov. and other phylogenetically related groups.

	***Group***	***Sequences***	***1***	***2***	***3***	***4***	***5***	***6***	***7***	***8***	***9***	***10***	***11***
***1***	**Leptolyngbyaceae I *(Thainema* gen. nov.)**	7											
***2***	Leptolyngbyaceae II (Prochlorotrichaceae)	21	**91.7**										
***3***	Merismopediaceae	3	**93.0**	90.6									
***4***	Thermosynechococcaceae	3	**92.0**	90.1	93.8								
***5***	Acaryochloridaceae	10	**93.1**	90.4	93.5	91.6							
***6***	Trichocoleaceae	10	**93.5**	91.5	93.8	92.6	92.6						
***7***	Pseudanabaenaceae I (*Toxifilum*)	4	**91.9**	91.8	91.9	91.6	91.3	94.1					
***8***	Leptolyngbyaceae	36	**91.5**	90.6	91.6	91.0	91.0	92.8	91.8				
***9***	Oculatellaceae	18	**91.5**	90.1	90.7	90.0	90.3	92.4	92.2	91.7			
***10***	Pseudanabaenaceae	19	**90.2**	88.9	89.4	89.9	89.5	90.8	89.1	89.1	89.7		
***11***	Gloeobacteraceae	1	**89.1**	88.0	89.2	89.5	88.3	88.9	88.5	88.0	88.2	88.4	

### Molecular diagnosis of families

**Fig 2:**
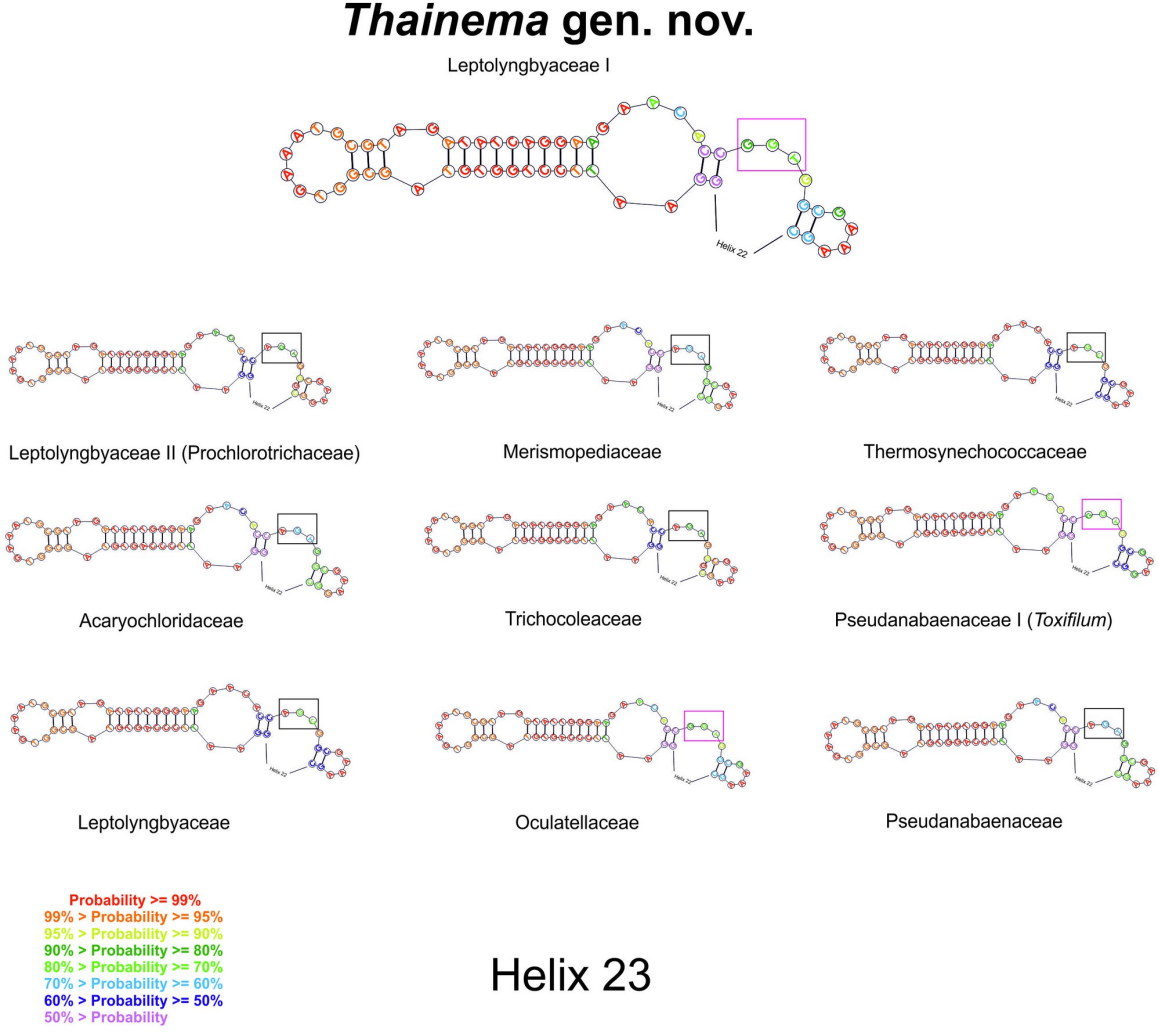
Helices 23 showing the molecular determinations of the proposed families.

**Fig 3:**
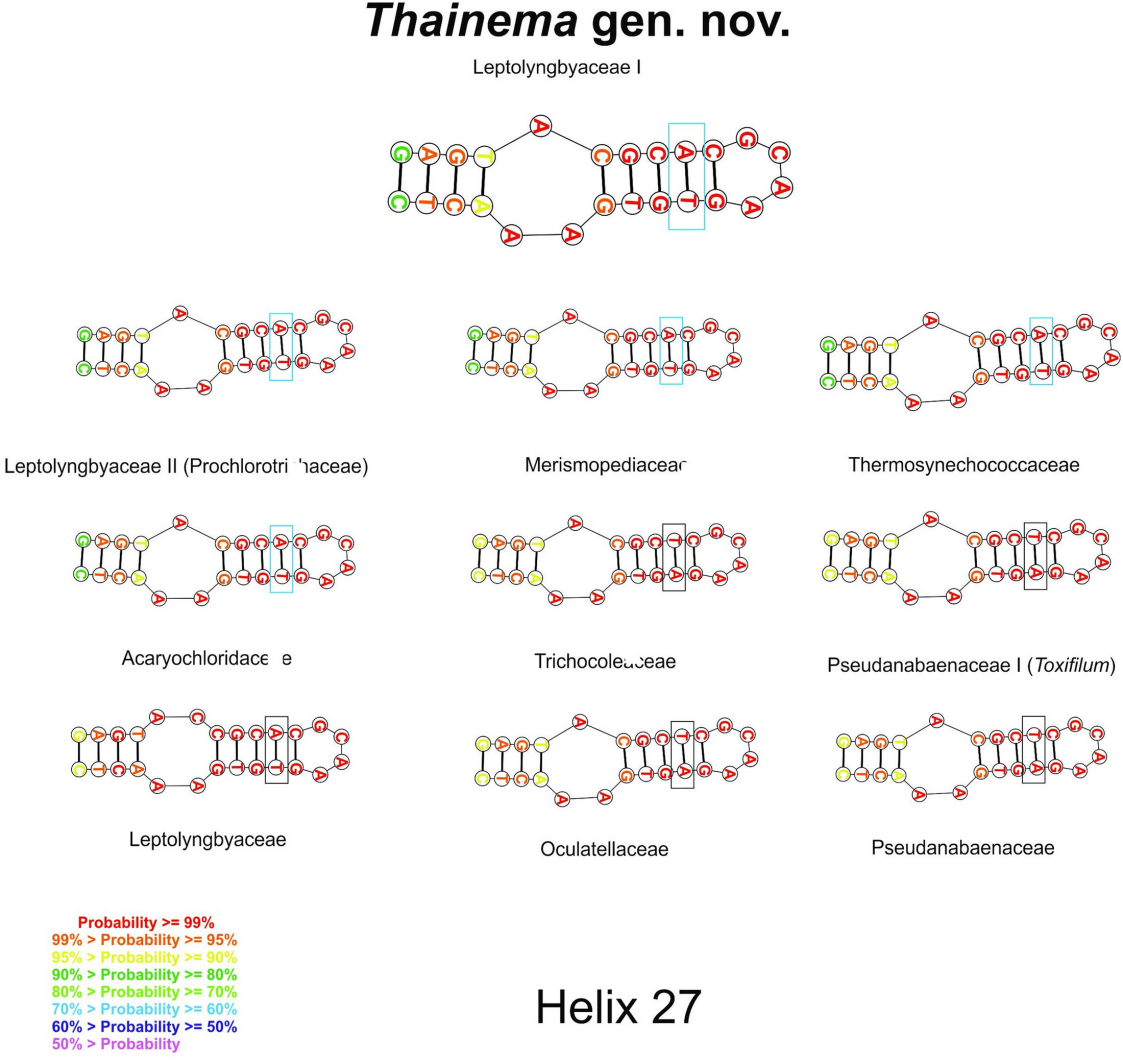
Helices 27 showing the molecular determinations of the proposed families.

The synapomorphic nucleotides in the 16S rRNA gene sequence in the different groups are listed in Table 3. The following groups were compared: 1) Leptolyngbyaceae I (*Thainema* gen. nov.); 2) Leptolyngbyaceae II (proposed by Mai et al. [14] as a member of the family Prochlorotrichaceae); 3) Merismopediaceae; 4) Thermoynechococcaceae; 5) Acaryochloridaceae; 6) Trichocoleaceae; 7) Pseudanabaenaceae I (*Toxifilum*, Zimba et al. [57]); 8) Leptolyngbyaceae; 9) Oculatellaceae; 10) Pseudanabaenaceae; and 11) Gloeobacteraceae. *Thainema* shared fewer similar nucleotides (helices 18 and 34) within the Pseudanabaenaceae family but more similar helices (18, 20, 27, and 34) within the Acaryochloridaceae family (Table 3). Additionally, the secondary structure of the 16S rRNA sequence (helices 23 and 27) in all families was examined for family-level distinction (Figs 2 and 3).

**Table 3:** Nucleotide variation between families within the order Synechococcales.

**Family**	**helix 18**	***Thainema* gen. nov**.
Leptolyngbyaceae1(Prochlorotrichaceae)	TGCCAGCAGCCGCGGTAA G A	
Merismopediaceae *(Aphanocapsa*)	TGCCAGCAGCCGCGGTAA G A	
Synechococcaceae *(Synechococcus*)	TGCCAGCAGCCGCGGTAA G A	
Acaryochloridaceae	TGCCAGCAGCCGCGGTAA G A	
Trichocoleaceae	TGCCAGCAGCCGCGGTAA G A	TGCCAGCAGCCGCGGTAA G A
Pseudanabaenaceae 1 (*Toxifilum*)	TGCCAGCAGCCGCGGTAA T A	
Leptolyngbyaceae	TGCCAGCAGCCGCGGTAA T A	
Oculatellaceae	TGCCAGCAGCCGCGGTAA T A	
Pseudanabaenaceae	TGCCAGCAGCCGCGGTAA G A	
	**helix 20**	
Leptolyngbyaceae1(Prochlorotrichaceae)	CTGAC G CT G AKGGACGAAA	
Merismopediaceae *(Aphanocapsa*)	CTGAC A CT C ATGGACGAAA	
Synechococcaceae *(Synechococcus*)	CTGAC A CT C ATGGACGAAA	
Acaryochloridaceae	CTGAC A CT C ATGGACGAAA	
Trichocoleaceae	CTGAC A CT G AKGGACGAAA	CTGAC A CT C ATGGACGAAA
Pseudanabaenaceae 1 (*Toxifilum*)	CTGAC A CT G AKGGACGAAA	
Leptolyngbyaceae	CTGAC A CT G AKGGACGAAA	
Oculatellaceae	CTGAC A CT G AKGGACGAAA	
Pseudanabaenaceae	CTGAC R CT G AKGGACGAAA	
	**helix 23**	
Leptolyngbyaceae1(Prochlorotrichaceae)	ATYRGGAAGAACACC A G T G	
Merismopediaceae *(Aphanocapsa*)	ATYRGGAAGAACACC A G T G	
Synechococcaceae *(Synechococcus*)	ATYRGGAAGAACACC A G T G	
Acaryochloridaceae	ATCGGGAAGAACACC A G T G	
Trichocoleaceae	ATCGGGAAGAACACC A G T G	ATCAGGAAGAACACC G G T G
Pseudanabaenaceae 1 *(Toxifilum*)	TTGGGAAGAACACCG G T G G	
Leptolyngbyaceae	ATTGGGAAGAACACC A G C G	
Oculatellaceae	ATTRGRAAGAACAYC G G T G	
Pseudanabaenaceae	ATCKGGAAGAACACC A G T G	
	**helix 27**	
Leptolyngbyaceae1(Prochlorotrichaceae)	GAGTACGC A CGCAAG T GTGAAACTC	
Merismopediaceae *(Aphanocapsa*)	GAGTACGC A CGCAAG T GTGAAACTC	
Synechococcaceae *(Synechococcus*)	GAGTACGC A CGCAAG T GTGAAACTC	
Acaryochloridaceae	GAGTACGC A CGCAAG T GTGAAACTC	
Trichocoleaceae	GAGTACGC T CGCAAG A GTGAAACTC	GAGTACGC A CGCAAG T GTGAAACTC
Pseudanabaenaceae 1 (*Toxifilum*)	GAGTACGC T CGCAAG A GTGAAACTC	
Leptolyngbyaceae	GAGTACGC A CGCAAG T GTGAAACTC	
Oculatellaceae	GAGTACGC T CGCAAG A GTGAAACTC	
Pseudanabaenaceae	GAGTACGG T CGCAAG A TTGAAACTC	
	**helix 34**	
Leptolyngbyaceae1(Prochlorotrichaceae)	CGTCAAGTCATCA T GCC C C	
Merismopediaceae *(Aphanocapsa*)	CGTCAAGTCATCA T GCC C C	
Synechococcaceae *(Synechococcus*)	CGTCAAGTCATCA T GCC C C	
Acaryochloridaceae	CGTCAAGTCAGCA T GCC C C	
Trichocoleaceae	CGTCAAGTCAGCA T GCC C C	CGTCAAGTCAGCA T GCC C C
Pseudanabaenaceae 1 (*Toxifilum*)	CGTCAAGTCATCA T GCC C C	
Leptolyngbyaceae	YGTCAAGTCAGCA T GCC C C	
Oculatellaceae	CGTCAAGTCAGCA T GCC C C	
Pseudanabaenaceae	CGTCAAGTCATCA T GCC C C	

The relevant nucleotides are shown in red.

### Species delimitation

**Fig 4:**
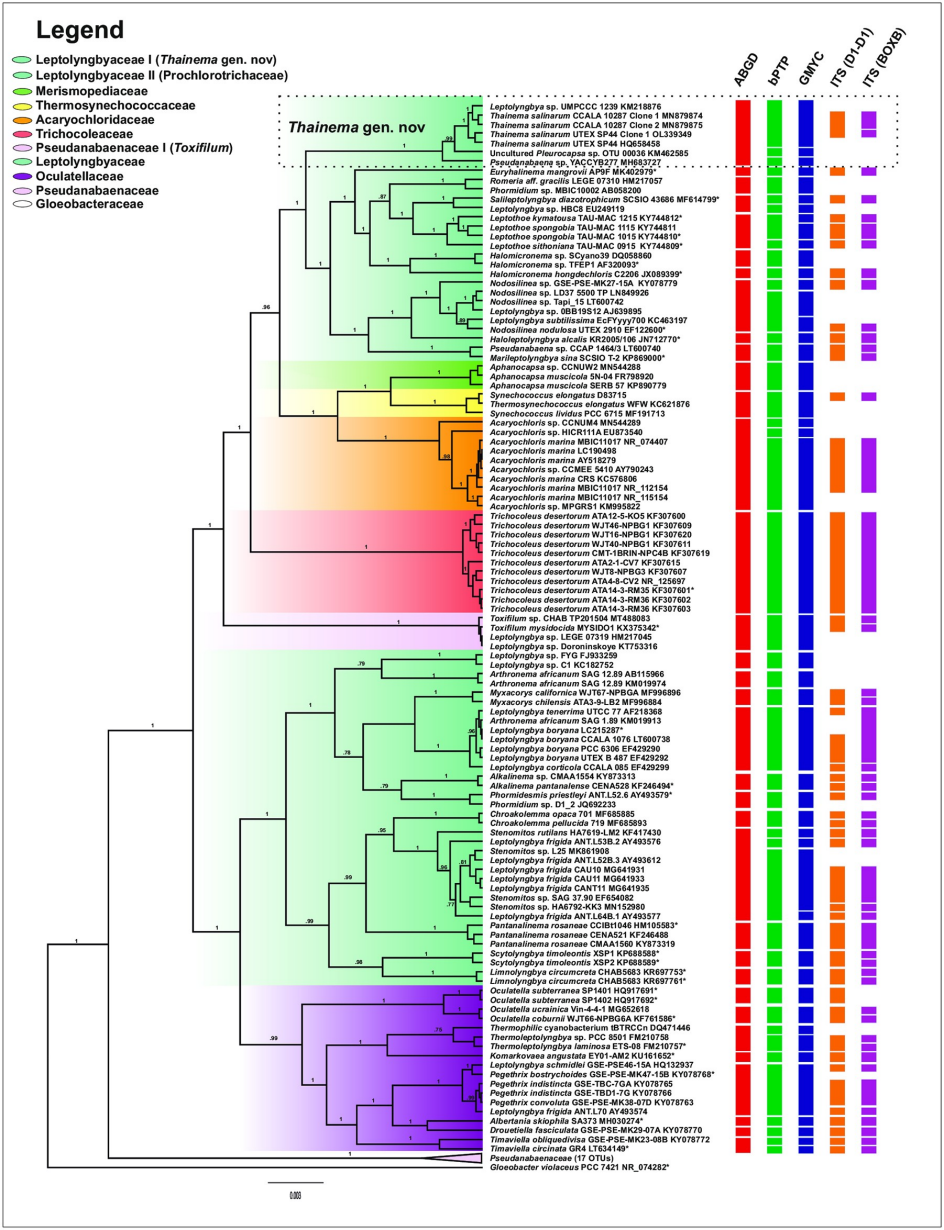
Phylogenetic tree reconstructed using Beast, based on the 16S rRNA gene sequence. The support values illustrate Bayesian posterior probabilities. Each column on the right shows a different species delimitation method, and each rectangle indicates a separate species. The legend indicates the proposed families belonging to the order Synechococcales.

The ABGD, bPTP, and GMYC analyses revealed 34, 47, and 54 species, respectively, based on the 16S rRNA sequences; however, 49 and 58 groups were recovered based on the D1-D1´ and Box-B helices of the 16S-23S rRNA ITS secondary structure, respectively (Fig 4 and S1 Fig).

The results of phylogenetic and species delimitation analyses revealed four additional strains closely related to *Thainema* strains: *Leptolyngbya* sp. UMPCCC 1239, which inhabited the coastal water of Sanibel Island, Florida, USA; *Pseudanabaena galeata* UTEX SP44, which originated from the pool sediment of Great Salt Plains, Oklahoma, USA; *Pseudanabaena* sp. YACCYB277, reported from Xinjiang, China; and uncultured *Pleurocapsa* sp. (Figs 1 and 4). In addition to the high similarity in the 16S rRNA gene sequences between members of *Thainema* gen. nov. (Table 1), uncultured *Pleurocapsa* sp. was an airborne organism from Oahu, Hawaii, USA, which grew on both marine and freshwater media; therefore, we could not exclude the possibility that it originated from almost the same type of habitat as the other strains in the new genus.

Only the ABGD analysis revealed the *Thainema* clade as a single species; however, the 16S rRNA threshold (sequence dissimilarity > 1.3%) provided strong evidence for a different species [58–60]. Species delimitation analyses (GMYC and bPTP) identified four and three species, respectively within *Thainema* gen. nov.

**Fig 5:**
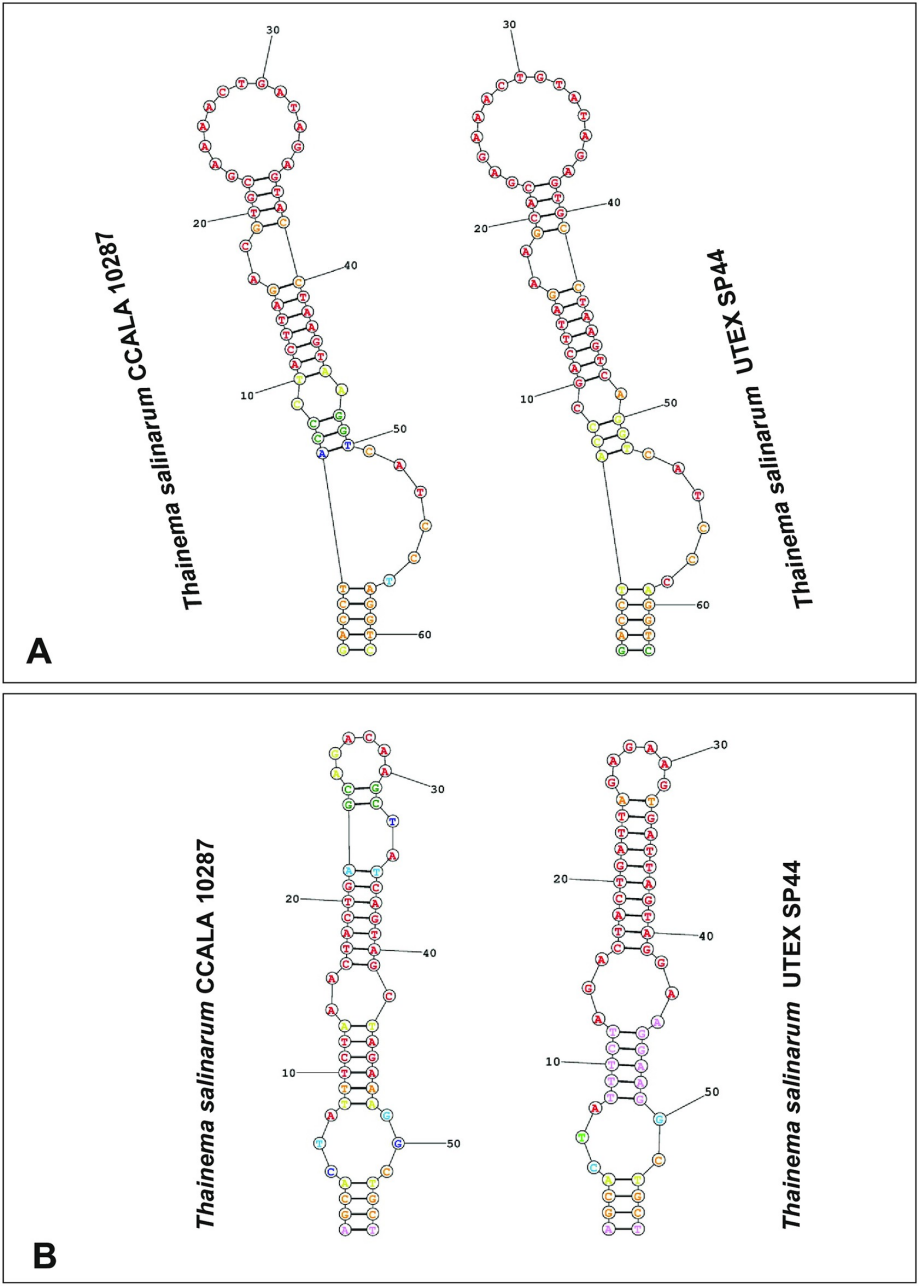
Secondary structure of the D1-D1´ and Box-B helices in the ITS region of strains CCALA 10287 and UTEX SP44. (A) D1-D1´ helices. (B) Box-B helices.

Additionally, secondary structures of D1-D1´ and Box-B helices in the ITS region of strains CCALA 10287 and UTEX SP44 are depicted in Fig 5. The D1-D1´ helices of strains CCALA 10287 and UTEX SP44 were highly similar (61 and 63 bp, respectively). By contrast, the Box-B helices (55 bp) of both strains differed at many positions (Fig 5). Sequences of the 16S-23S ITS region in the other strains were not available for comparison.

### Morphological and taxonomic descriptions

The previous description of the strain *Thainema*, which was introduced as *Halomicronema* sp. [39], did not include some of the morphologic features, such as colorless sheath, granules, and the size of trichomes was only approximated. In addition, authors of the cited work decided not to establish a new species. Here, we amend the description of the species below to show its separation from the genus *Halomicronema*, based on both morphological and molecular characterizations. We describe one new genus that includes one new species, whose monophyletic position is strongly supported.

*Class Cyanophyceae*

*Order Synechococcales*

*Family Leptolyngbyaceae*

***Thainema*** Rasouli-Dogaheh et Hauer **gen. nov**.

#### Description

**Fig 6:**
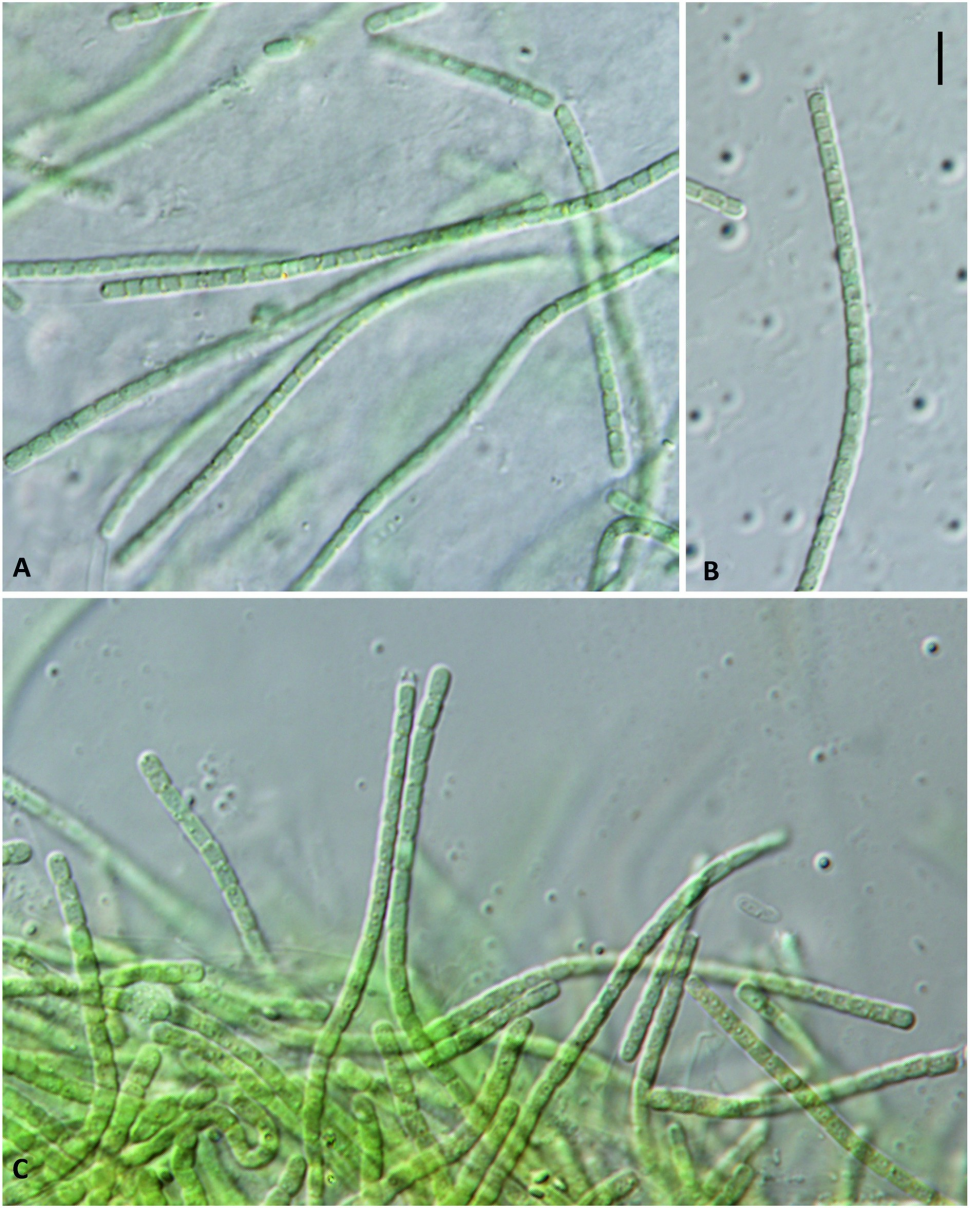
Light microscope view of *Thainema salinarum*. The golden granules at cross-walls and sheaths are observed. Scale bar = 10 μm.

**Fig 7:**
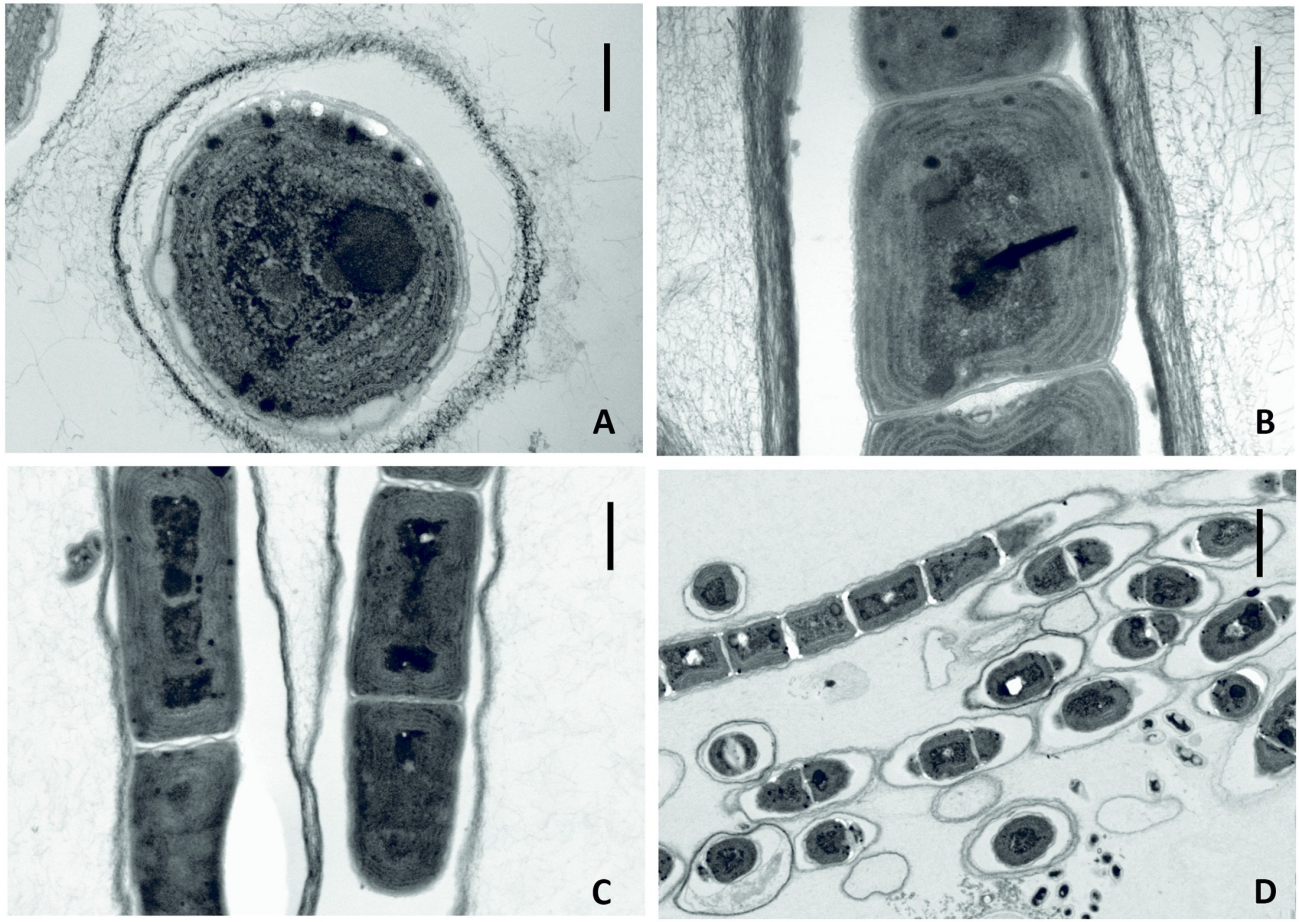
Micrographs of *Thainema salinarum* obtained with a transmission electron microscope (TEM). (A, B) Cells with parietal thylakoids, granules, and sheath. Scale bar = 0.5 μm. (C) Longitudinal section of filaments showing the presence of parietal thylakoids. Scale bar = 1 μm. (D) Short filament showing a round apical cell. Scale bar = 5 μm.

Filaments straight or flexuous, mostly bright blue-green or green; no branching, immotile; sheath colorless, sometimes indistinct under the LM; no heterocytes or other special cells present. Trichomes solitary, cylindrical, <3 μm wide, and slightly constricted at the cross-walls. Cells mostly isodiametric or slightly longer than wide, usually with pale-to-shiny granules located at cross-walls; no aerotopes. Terminal cells rounded, without calyptra, and morphologically similar to other cells. Reproduction by trichome fragmentation. Halophilic to halotolerant (Figs 6 and 7).

#### Etymology

Thai (ไทย) refers to Thailand—country of origin of the type species,—nema (νήμα)—thread (Gr.), refers to appearance of the organism.

#### Type species

***Thainema salinarum*** Rasouli-Dogaheh et Hauer, **sp. nov**.

#### Description

Colony pale to bright blue-green. Filaments, 1–3 μm wide. Trichomes mostly straight and slightly constricted, or not constricted at the cross-walls. One trichome present in a sheath with no branching, but the presence of sheaths for some filaments could not be determined with certainty under the LM. Cells longer than wide (2–3.5 μm long), mostly isodiametric (1.5–2 μm long) with pale yellowish-to-golden granules. Reproduction by trichome fragmentation. Aerotopes absent. Terminal cells typically rounded, without calyptra. Necridic cells absent (Fig 6). TEM micrographs demonstrated definite presence of sheaths. Parietal thylakoids (4–5 per cell) were visible in the longitudinal section but were less discernable in the cross-section. Cyanophycin granules were observed among thylakoids. Apical cells were rounded. The cell wall was simple like that of most species in the Leptolyngbyaceae family (Fig 7).

#### Etymology

Name of the species refers to the original habitat of the material.

#### Type locality

Thailand, Petchaburi Province, Ban Laem District (13.30 N, 100.07 E). Mixed with other cyanobacteria in green filamentous mats on a wet soil surface, where the salinity of ponds ranged from 90 to 250 parts per thousand [39].

#### Holotype here designated

CBFS A-119-1 at Herbarium of the University of South Bohemia, České Budějovice, Czech Republic.

#### Reference strain

CCALA 10287 at the CCALA culture collection, Institute of Botany AS CR, Třeboň, Czech Republic.

#### Diagnosis

According to a recent taxonomic classification [9], *Thainema salinarum* differs from *Halomicronema excentricum* morphologically in terms of cell size and the lack of gliding motility and gas vesicles. However, both species share the same ecological habitat and were isolated from benthic microbial mats in a manmade hypersaline environment.

## Discussion

The multilocus and 16S rRNA data-based phylogenetic trees revealed a new monophyletic genus, *Thainema* gen. nov. Our results demonstrate that *Thainema* gen. nov. is a sister clade of the genera *Nodosilinea* and *Halomicronema*, and was previously proposed to be transferred from the Leptolyngbyaceae family to Prochlorotrichacae [14]. It is particularly problematic to delimit a family in prokaryotes based on their morphological characteristics [61, 62]. In cyanobacteria, a family has traditionally been described by a set of morphological features, which do not reflect its evolutionary history [9, 63]. Diverse morphological autapomorphy was one of the greatest restrictions inside several genera within different families, especially in the order Synechococcales. Therefore, taxonomists discovered molecular markers in the amino acid and 16S rRNA gene sequences of organisms, which were shared only by members of a specific population of species, and these shared sequences denote synapomorphies between the common ancestors and all of their descendants [64–67]. Additionally, the secondary structure of 16S rRNA has been recognized as a useful tool for taxonomic classification in the higher levels of cyanobacteria [55]. According to the latest research (e.g. [14]), measurement of morphological characteristics and determination of 16S rRNA genetic identity cutoff values, synapomorphic nucleotides of the 16S rRNA sequence, and secondary structures of helices 18, 20, 23, 27, and 34 could lead to the family-level identification of a species. Most of the previous studies suggested further division of the Leptolyngbyaceae family [19, 29, 30, 35, 36, 38]. Recently, Mai et al. [14] proposed that the Leptolyngbyaceae should be broken down into four family-level clades (Prochlorotrichaceae, Oculatellaceae, Leptolyngbyaceae, and Trichocoleaceae). In the current study, it was not feasible to use the 16S rRNA genetic identity cutoff values for family delimitation because of high average similarity (92.1%) between genera belonging to different families, which is consistent with the result of Mai et al. [14]. However, *Thainema* gen. nov. shared the highest number of nucleotides with the Acaryochloridaceae family members at conserved sites in the 16s rRNA sequence.

In this study, both 16S rRNA and multilocus datasets revealed the same phylogenetic relationships between clades (Figs 1 and 4), which is consistent with previous studies [14, 57, 68, 69]. Even though previous studies [19, 31, 32] had only considered the genus *Leptolyngbya* as a polyphyletic genus within the family Leptolyngbyaceae, our phylogenetic trees revealed the Leptolyngbyaceae family as a polyphyletic group, and this classification is congruent with that of Mai et al. [14], who based their classification on the sequence of *rpoC1*. However, the family Leptolyngbyaceae has received much greater attention from both traditional and modern taxonomy, than any other families [70]. Therefore, a precise taxonomic analysis was required to re-evaluate its phyletic status. Consequently, we avoided proposing a new family or placing the new genus into the Acaryochloridaceae family; instead, we present *Thainema* gen. nov as a member of the Leptolyngbyaceae family. Alternatively, we suggest a hybrid approach, which incorporates all previously submitted methods as well as the chemical composition used by Zimba et al. [57], for family-level identification of the *Toxifilum* genus within the Pseudanabaenaceae family for future studies.

In this work, we sought to identify the molecular and morphological overlap between the CCALA 10287 strain (isolated from wet soil in shallow evaporation basins in Ban Laem district, Petchaburi Province, Thailand [39]) and four other identical strains not related to any of the recognized Leptolyngbyaceae taxa. The public sequence repositories contain a large number of sequences that have either been incorrectly assigned to known organisms or have been assigned a provisional taxonomic designation, which is a threat to cyanobacterial taxonomy. The NCBI Reference Sequences (RefSeq) Database (https://www.ncbi.nlm.nih.gov/refseq/) can be used as a tool for the identification of reference sequences, but it has some limitations. The current version of RefSeq follows neither the International Code of Nomenclature for Algae, Fungi, or Plants (ICN) [71], nor the International Code of Nomenclature of Prokaryotes (ICNP) [72], both of which are used for the classification of cyanobacteria. This problem can lead to incorrect phylogenies or overlooked novel taxa. Despite the vast amount of molecular data available from all over the world, many taxa remain unrecognized. For example, Blank & Hinman [73] isolated 29 strains and performed phylogenetic analyses based on their 16S rRNA gene sequences. Most of the cyanobacterial strains clustered in four large, well-characterized clades, but some strains such as *Leptolyngbya* sp. UMPCCC 1239 remained separate and were submitted to NCBI as an unclassified sequence. In the current study, we included such sequences (KM218876, MH683727, and KM462585) in our analyses to determine their phylogenetic placement and confirmed that it belongs to a new taxon in the family Leptolyngbyaceae. Thus, our results will assist in reducing the number of sequences with provisional names that belong to currently unknown taxa. Overall, it is highly advisable to utilize as many sequences of type species (according to ICN) or type strains (according to ICNP) and other reference sequences of particular taxa published in their protologues or taxonomic revisions as possible. Such sequences are listed, for example, in CyanoDB 2.0 [70].

In the present study, data available on the origins of the studied strains show an interesting pattern in their geographical distribution. Despite substantial molecular analyses conducted to expand our knowledge of cyanobacterial diversity worldwide in saline, non-planktic habitats, including marine coasts (e.g., [74, 75]), mangroves (e.g., [69]), and brackish waters (e.g., [76]), and the high number of publicly available sequences, the distribution of *Thainema* gen. nov. seems limited to the Northern Hemisphere, notably East Asia (Thailand, this study, red circle on the map; China, accession number: MH683727), the Pacific Region (Hawaii, accession number: KM462585), and North America (strains UMPCCC 1239 and UTEX SP44 [red circle on the map], accession numbers: KM218876 and HQ658458, respectively). This shows that the Baas Becking (1934) hypothesis, i.e., “everything is everywhere, but the environment selects”, cannot generally be applied to all microorganisms. We speculate that the taxon originally evolved in North America, and its propagules were then transported by trade winds to Asia. Sherwood et al. [77], who investigated airborne algae on the island of Oahu, Hawaii, and published sequence KM462585, supports this hypothesis. On the generic level, the distribution pattern of cyanobacterial strains is plainly in contrast to that of either the cosmopolitan taxa, such as members of the genus *Nostoc* Vaucher ex Bornet *et* Flahault ([78]: 181), or *Myxacorys* Pietrasiak et Johansen ([79]: 980), whose distribution is limited to the deserts of the Western Hemisphere. In many cases, the distribution pattern can be skewed by the disproportionate activities of researchers around the world, since studies are often performed in the “favorite research locations” of specific teams. We believe that such a bias is low in saline and non-planktic habitats.

According to the literature on species concepts [8, 80, 81], it seems that the classification of genera, at least in certain cases, is more complicated than the separation of different species within the same genus [14]. Furthermore, employing the 16S rRNA gene is not adequate for species delimitation of all genera of the cyanobacteria [31, 82]. The 16S-23S ITS region can be better applied to species separation [19–21, 23]. Here, the question that arises is whether all strains in the newly described clade (*Thainema*) belong to the same species, or whether they should be considered as separate taxa. The main taxonomic approaches used in this study for the classification of *Thainema* provided consistent morphological, biogeographical, ecological, and molecular data within the genus. Therefore, we followed 16s rRNA-based species delimitation approaches [83–89] as well as utility of secondary structure in the 16S-23S ITS region [30, 82, 90], which both are often used to separate genetically distinct taxa.

Hence, P-distance-based methods, such as ABGD and 16S rRNA sequence similarity, were used in this study. The genetic distance approach has been one of the most commonly used methods for the identification of eukaryotic species [91–93]; however, it has also been used for prokaryotes such as cyanobacteria [94]. In this study, both analyses confirmed that the *Thainema* lineage diverged from other lineages of cyanobacteria (Fig 4, Table 2). In addition, the results of ABGD analysis revealed that the *Thainema* clade represents a single species; however, considering the length of sequences, the sequence similarity threshold separated the clade into four species. While the ABGD approach is promising, it has certain disadvantages when used for cyanobacterial species delimitation [94]. Our results showed that this method is not well matched with the threshold of similarity (97% or 99%) in genetic distances. Thus, it may not be the best method for determining gaps among multiple species, based on nucleotide substitutions of a single gene [94], especially when the species are genetically polyphyletic [95]. Therefore, employing additional methods for delimiting the species units would lead to a more accurate classification.

In this study, we used tree-based methods (GMYC, bPTP) on the 16S rRNA dataset [96–99], and found that the results of the similarity method were almost in accord with those of tree-based analyses. Dvořák et al. [100] only used the PTP method, and proved that it could adequately delimit the cyanobacterial species. In the current study, the GMYC approach was more adept at recognizing species than the bPTP and ABGD approaches. This result is consistent with previous findings, which showed that the GMYC method provided more usable taxonomic units than other species delimitation methodologies [91, 92, 97, 99, 101–103]. Furthermore, one functional pattern analysis (ITS secondary structure) of the 16S-23S ITS dataset was applied in this study. This analysis clearly represented diagnostic apomorphic characteristics, which were consistent with the 16S rRNA gene-based phylogeny of cyanobacteria [19, 20, 104]. Unfortunately, we could not gain access to all molecular data on the 16S-23S ITS region. Nevertheless, among the various methods used in this study, the16S-23S ITS secondary structure proved to be the most sensitive tool for separating the different genetic groups of cyanobacteria. Moreover, we did not have morphological data to reliably confirm the existence of more species in the newly described genus. Because of the above-mentioned factors, we hesitate to establish more than one species in the genus based on the current dataset, we also recommend using an integrating species delimitation method and the 16S-23S ITS secondary structure to develop an accurate taxonomic workflow for future research.

## Supporting information

S1 Fig Phylogenetic tree reconstructed using Beast, based on the 16S rRNA gene sequence. The support values illustrate Bayesian posterior probabilities. Each column on the right shows a different species delimitation method, and each rectangle indicates a separate species. The legend indicates the proposed families belonging to the order Synechococcales.
(TIF)

S1 Table Primers used for PCR amplification. (DOCX)

S2 Table PCR protocol used in this study. (DOCX)

S3 Table Dataset with access information. List of all sequences, accession numbers for 16S rRNA, 16S-23S ITS, *rpoC1* and *rbcLX* genes, length and the authors of the sequences used in the phylogenetic trees. Reference sequences of taxa marked with an asterisk (*). The names of accession numbers without the asterisk might not be accurate names.
(XLSX)
