# Supplementary figures and images for: Thainema gen. nov. (Leptolyngbyaceae, Synechococcales): A new genus of simple trichal cyanobacteria isolated from a solar saltern environment in Thailand
Source: PLoS One. 2022 Jan 7;17(1):e0261682. doi: 10.1371/journal.pone.0261682 (PMC8741055; doi:10.1371/journal.pone.0261682)

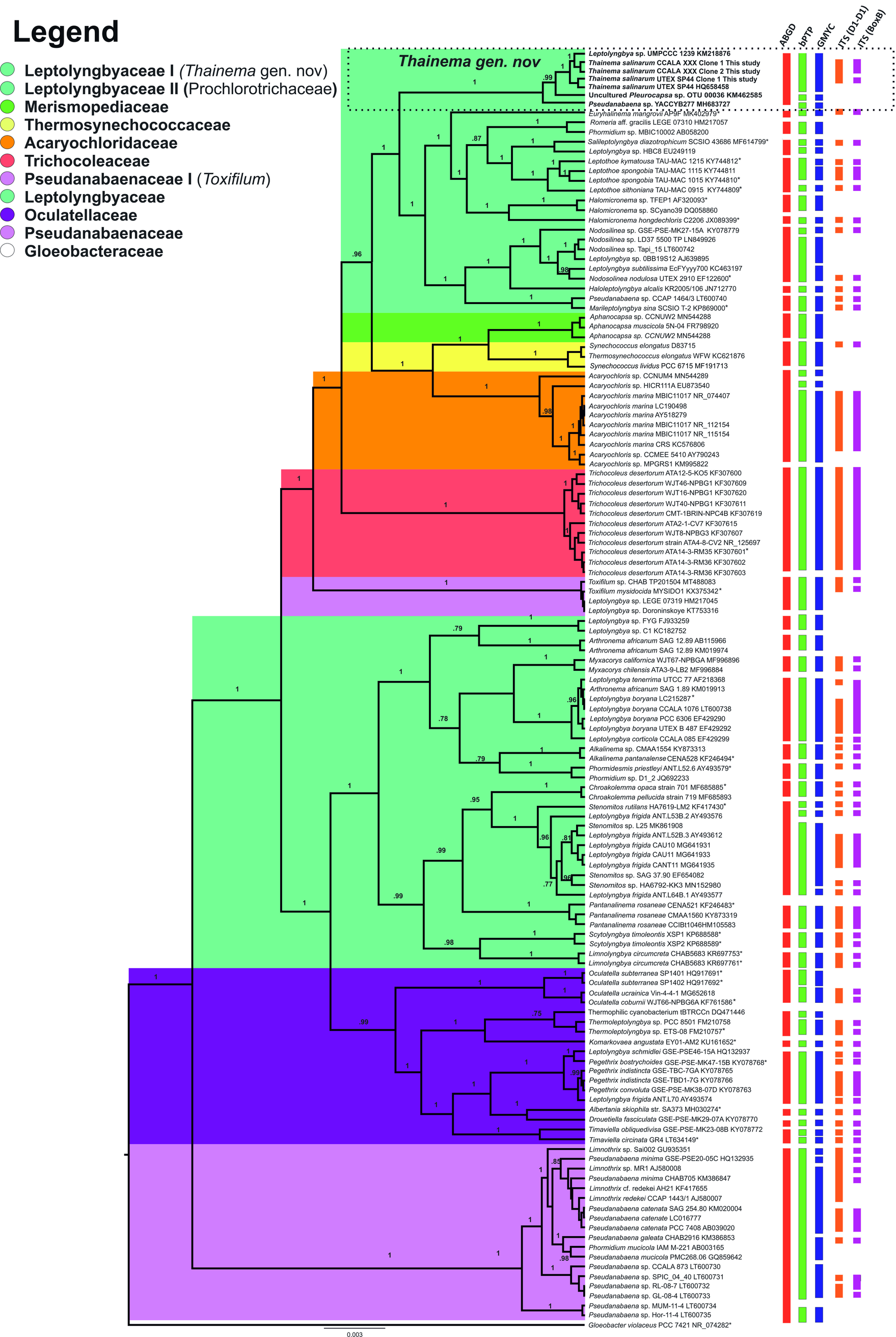

Supplement: S1 Fig — The support values illustrate Bayesian posterior probabilities. Each column on the right shows a different species delimitation method, and each rectangle indicates a separate species. The legend indicates the proposed families belonging to the order Synechococcales. (TIF) [file pone.0261682.s001.tif]
