# Supplementary material for: Thainema gen. nov. (Leptolyngbyaceae, Synechococcales): A new genus of simple trichal cyanobacteria isolated from a solar saltern environment in Thailand
Source: PLoS One. 2022 Jan 7;17(1):e0261682. doi: 10.1371/journal.pone.0261682 (PMC8741055; doi:10.1371/journal.pone.0261682)
Supplement: S1 Table — (DOCX) [file pone.0261682.s002.docx]

| Primers | Sequence (5'-3') | Source |
| --- | --- | --- |
| 16S27F | AGA GTT TGA TCC TGG CTC AG | Wilmotte et al. 1993 |
| 23S30R | CTT CGC CTC TGT GTG CCT AGG T | Taton et al. 2003 |
| rpc/MF | GGT GAR GTN ACN AAR CCA GAR AC | Seo & Yokota 2003 |
| rpc/CR | CCA GAR TAG TCN ACC CGT TTA CC | Seo & Yokota 2003 |
| CW | CGT AGC TTC CGG TGG TAT CCA CGT | Rudi et al. 1998 |
| CX | GGG GCA GGT AAG AAA GGG TTT CGT A | Rudi et al. 1998 |
