# Supplementary material for: Thainema gen. nov. (Leptolyngbyaceae, Synechococcales): A new genus of simple trichal cyanobacteria isolated from a solar saltern environment in Thailand
Source: PLoS One. 2022 Jan 7;17(1):e0261682. doi: 10.1371/journal.pone.0261682 (PMC8741055; doi:10.1371/journal.pone.0261682)
Supplement: S2 Table — (DOCX) [file pone.0261682.s003.docx]

| **No.** | **PCR step** | **16s rRNA-ITS** | | **rpoC1** | | **rbcLX** | |
| --- | --- | --- | --- | --- | --- | --- | --- |
|  |  | **Temp.** | **Time Repeat cycle** | **Temp.** | **Time Repeat cycle** | **Temp.** | **Time Repeat cycle** |
| 1 | Initial denaturation | 94 °C | 5 min | 95 °C | 3 min | 94 °C | 5 min |
| 2 | Denaturation | 94 °C | 30 sec | 95 °C | 30 sec | 94 °C | 40 sec |
| 3 | Annealing | 54 °C | 30 sec | 51°C | 30 sec | 52 °C | 1 min |
| 4 | Extension | 72 °C | 90 sec | 72 °C | 1 min | 72 °C | 100 sec |
| 5 | Final extension | 72 °C | 10 min | 72 °C | 10 min | 72 °C | 10 min |
| 6 | Cooling | 15 °C | --- | 15 °C | --- | 15 °C | --- |
| Step 2 to 4 were repeated 45 times | | | | | | | |
